# Predictors of prognosis in children under six years of age with recurrent croup: a retrospective cohort study

**DOI:** 10.1186/s12887-026-06894-4

**Published:** 2026-04-27

**Authors:** Adem Yaşar, Özge Yılmaz, Mustafa Eres, Atike Açalya Tatar, Hasan Yüksel

**Affiliations:** 1 School of Medicine, Department of Pediatric Allergy and Immunology, Manisa Celal Bayar University, Manisa, Türkiye; 2 School of Medicine, Department of Pediatric Allergy and Immunology, and Pediatric Pulmonology, Manisa Celal Bayar University, Manisa, Türkiye

**Keywords:** Recurrent croup, Pediatric airway obstruction, Atopy, Flexible bronchoscopy, Gastroesophageal reflux

## Abstract

**Background:**

Croup is a common cause of upper respiratory tract obstruction in young children. Although most cases resolve spontaneously, some children experience recurrent attacks. Identifying prognostic factors that determine the risk of recurrence in children with recurrent croup may help optimize follow-up and treatment strategies.

We aimed to investigate the clinical, demographic, and laboratory characteristics of children under six years of age with recurrent croup and to identify independent predictors of disease recurrence during a 6- and 12-month follow-up period.

**Methods:**

We conducted a retrospective cohort study of 69 children (<6 years) diagnosed with recurrent croup at a single tertiary pediatric pulmonology and allergy–immunology center. Eligible subjects (≥2 clinician-documented episodes within 12 months) presented between October 2020 and October 2023 and had at least 12 months of follow-up. We gathered information from medical records about the children's background, health details, laboratory results, and treatments. Binary logistic regression was used to identify independent predictors of croup recurrence within the first 6 and 12 months.

**Results:**

The mean age was 4.4±1.5 years, and 67% (*n*=46) were male. Within 6 months, 40.6% of cases experienced at least one recurrent attack, and 11.6% experienced a croup attack between 6 and 12 months. For 6-month recurrence, a family history of atopy (OR: 7.50, 95% CI: 1.96–28.68; *p*=0.003) and a higher number of croup attacks before follow-up (OR: 1.43, 95% CI: 1.06–1.92; *p*=0.020) were independent predictors, but age and cough trigger were not significant. For 12-month recurrence, family history of atopy (OR: 9.11, 95% CI: 1.12–74.00; *p*=0.039) and the number of attacks prior to follow-up (OR: 1.66, 95% CI: 1.08–2.54; *p*=0.020) were significant, while advanced age emerged as a protective factor (OR: 0.48, 95% CI: 0.24–0.95; *p*=0.035). In a small, clinically selected subgroup, flexible optical bronchoscopy and thoracic CT angiography identified airway compression and laryngeal findings possibly consistent with reflux-related irritation.

**Conclusions:**

In young children with recurrent croup, a family history of atopy and the number of prior croup episodes were independently associated with a higher probability of further recurrences during 6- and 12-month follow-up. These easily available clinical factors may help identify children who warrant closer follow-up, but validated risk thresholds and diagnostic pathways require prospective external validation.

## Background

Croup is a common respiratory tract disease in young children and is primarily caused by viral infections, particularly parainfluenza virus types 1 and 3, which cause inflammation and swelling in the upper respiratory tract [1]. It usually affects children between six months and six years of age, with the highest incidence occurring at two years of age [2]. Clinically, croup is characterized by a distinct “barking” cough that worsens at night, inspiratory stridor, hoarseness, and varying degrees of respiratory distress [3]. Most cases resolve spontaneously or with treatment, but severe cases requiring hospitalization occur when symptoms cause significant respiratory difficulties [4].

Croup usually resolves on its own; however, in some cases, attacks may recur two or more times within a year, suggesting the presence of underlying variables that affect the recurrence and severity of the disease [5]. Recurrent croup may signify underlying physical anomalies, immunological deficiencies, or environmental exposures, including tobacco smoke, allergens, or air pollution; thus, physicians must ascertain these factors [6]. Furthermore, conditions such as gastroesophageal reflux disease and atopy have been associated with recurrent cases, suggesting a multifactorial etiology [7–9].

Despite the clinical significance of recurrent croup and its relatively high prevalence, there is a lack of comprehensive studies in the literature identifying prognostic indicators that predict the likelihood of recurrence. Significant gaps in knowledge persist regarding the effect of clinical, demographic, and laboratory variables, either alone or in combination, on the likelihood of recurrent attacks [10]. Recent studies have investigated the neutrophil-lymphocyte ratio, C-reactive protein, and eosinophilic markers to distinguish severe and recurrent croup cases; however, definitive conclusions have not been reached [11]. Furthermore, advancements in artificial intelligence and predictive modeling exhibit the potential to amalgamate various clinical and laboratory variables to pinpoint high-risk cases of recurrent croup; however, these methodologies necessitate substantial clinical data to ascertain which markers are most indicative, underscoring the necessity for extensive studies like the current one [12].

This retrospective cohort study aims to address these gaps by evaluating children under six years of age with recurrent croup, focusing on clinical, demographic, and laboratory variables that influence croup recurrence. Our objective is to systematically analyze and identify reliable prognostic indicators for early recurrence in cases diagnosed with recurrent croup over a 6- to 12- month follow-up period. This could facilitate advanced risk stratification and personalized care approaches for high-risk pediatric groups and provide a foundation for future predictive models.

## Methods

### Study design, ethics approval and consent to participate

This retrospective cohort study was approved by the Manisa Celal Bayar University School of Medicine, Health Sciences Ethics Committee (Approval No: 03.04.2024/20.248.486/2343). All methods were performed in accordance with the Declaration of Helsinki and relevant national regulations. Given the retrospective design, use of de-identified data, minimal risk to participants, and impracticability of obtaining consent, the Ethics Committee waived the requirement for written informed consent from participants/their legal guardians.

### Case selection and data collection

In this retrospective cohort study, we reviewed our institution’s electronic medical records. We identified children aged 0–6 years who visited the pediatric pulmonology or pediatric immunology and allergy outpatient clinics. The study included cases with two or more documented episodes of croup by a clinician between October 2020 and October 2023. To ensure complete data collection, we included cases that had been followed up regularly for at least 12 months or more.

Cases with craniofacial anomalies, chronic lung diseases (e.g., primary ciliary dyskinesia, cystic fibrosis, clinically significant bronchopulmonary dysplasia) or known immunodeficiencies were excluded from the study.

### Clinical and demographic data collection

Demographic data collected from case files include age, gender, gestational age, mode of delivery, and history of admission to the neonatal intensive care unit (NICU). Clinical variables include the number of previous croup attacks before the initial examination, nighttime cough, cough during activity, type of stridor, wheezing, previous upper respiratory tract infections (URTI), nighttime feeding, gastroesophageal reflux symptoms, and symptoms of atopic disease (asthma, allergic rhinitis, food allergy, etc.) were included. Family history of asthma or allergic disease and maternal atopy were recorded. Environmental exposures such as passive smoking exposure and type of indoor heating were also recorded. All personal identifiers were anonymized in accordance with data protection policies.

### Laboratory and treatment data

Laboratory parameters obtained from medical records included complete blood count, peripheral eosinophil count, and total serum IgE levels. These tests were performed as part of the initial evaluation at our clinic; when multiple measurements were available, the earliest value obtained within a predefined time interval related to the index visit was used. Additionally, skin prick tests (SPT) for aeroallergens were also recorded. During follow-up, treatment strategies, including the use of inhaled corticosteroids, nasal corticosteroids, anti-reflux medications, and antihistamines, were documented. Personalized treatment plans based on clinicians’ decisions were recorded in the files.

### Flexible bronchoscopy and radiological evaluation

Flexible optical bronchoscopy (FOB) was performed in patients with persistent or atypical symptoms. Findings such as posterior laryngeal erythema, interarytenoid edema, increased secretions, or signs of airway compression were recorded. Additionally, when structural abnormalities were suspected, chest computed tomography (CT) and chest CT angiography were examined for confirmation.

### Follow-up and outcome definition

Patients diagnosed with recurrent croup were followed up for 12 months from the date of initial presentation. Croup recurrence was defined as any new episode fulfilling the clinical criteria for croup (barking cough with or without inspiratory stridor and hoarseness) that was documented by a pediatrician in our outpatient clinic or emergency department and treated with systemic corticosteroids with or without nebulized therapy. Episodes were classified as occurring within the first 6 months or between 6 and 12 months from the date of the index visit.

### Statistical analysis

Statistical analyses were performed using SPSS version 22. Descriptive statistics were reported using means and standard deviations for continuous variables that showed a normal distribution. Categorical variables were presented as frequencies and percentages. Group comparisons were performed using the Student t-test for continuous variables and the chi-square test for categorical variables. Binary logistic regression analysis was performed to identify independent predictors of recurrence within the first 6 and 12 months. Variables with clinical significance or a p-value < 0.20 in univariate analysis were included in the multivariate model. Odds ratios (OR) with 95% confidence intervals (CI) were calculated. Model fit was assessed using the Akaike Information Criterion (AIC) and McFadden’s pseudo R² value; lower AIC values indicate a better balance between goodness-of-fit and model complexity, whereas higher McFadden’s pseudo R² values reflect better overall model performance.

## Results

### Socio-demographic and clinical characteristics

**Fig. 1:**
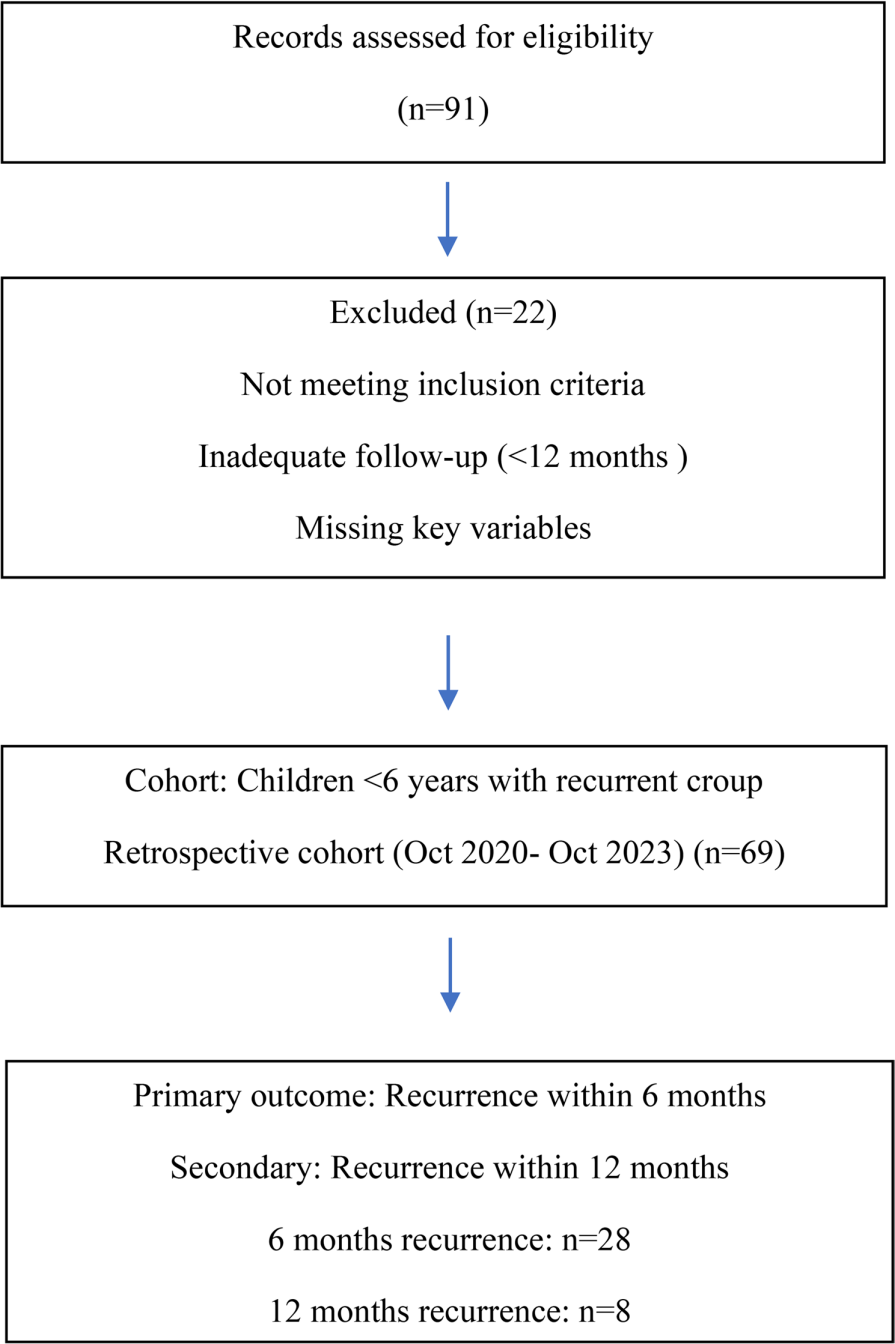
STROBE flow diagram of eligibility, exclusions, inclusion, and outcome ascertainment for the recurrent croup cohort (October 2020–October 2023).

Of the 91 cases presenting with clinician-confirmed and documented recurrent croup between October 2020 and October 2023, 69 cases met the inclusion criteria (Fig. 1). Data from 69 cases followed for ≥ 12 months were analyzed. The mean age of the patients was 4.42 ± 1.49 years. 67% of patients (*n* = 46) were male. Regarding birth history, 38% of patients (*n* = 26) were born via normal spontaneous vaginal delivery (NSVD) and 91% (*n* = 63) were born at term. A history of admission to the pediatric intensive care unit (PICU) prior to enrollment was reported in 10% (*n* = 7). In addition, 3% (*n* = 2) had a remote neonatal history of mild bronchopulmonary dysplasia without ongoing chronic lung disease at the time of study inclusion. The mean number of croup attacks prior to enrollment was 3.96 ± 2.04. During the first 6 months of follow-up, the mean number of croup attacks was 0.71 ± 1.11, and it decreased to 0.12 ± 0.32 in the first 12 months.

### Laboratory and clinical characteristics

Laboratory findings showed that the mean peripheral blood eosinophil count was 299 ± 314 cells/µL and the mean serum total IgE level was 180 ± 408 IU/mL. In addition, 23% of patients (*n* = 16) had a positive skin prick test result for aeroallergens.

Factors that could influence the clinical course were also evaluated. Nighttime cough was observed in 55% of cases (*n* = 38), and cough triggered by exertion was observed in 21% (*n* = 15). A previous upper respiratory tract infection (URTI) was present in 37% of cases (*n* = 26). Environmental exposures were common: 52% of patients (*n* = 36) had nighttime feeding habits, and 36% (*n* = 25) were exposed to indoor tobacco smoke. Regarding home heating methods, 58% (*n* = 40) used air conditioning, 27.5% (*n* = 19) used central heating (radiators), and 14.5% (*n* = 10) used stove heating.

Among comorbid allergic conditions, allergic rhinoconjunctivitis was detected in 33% (*n* = 23) and atopic dermatitis in 5% (*n* = 3). Wheezing episodes were reported in 20% (*n* = 13) of patients. 26% (*n* = 18) had a family history of atopic disease, and 42% (*n* = 29) had a family history of asthma.

### Treatment approaches applied during follow-up

**Fig. 2:**
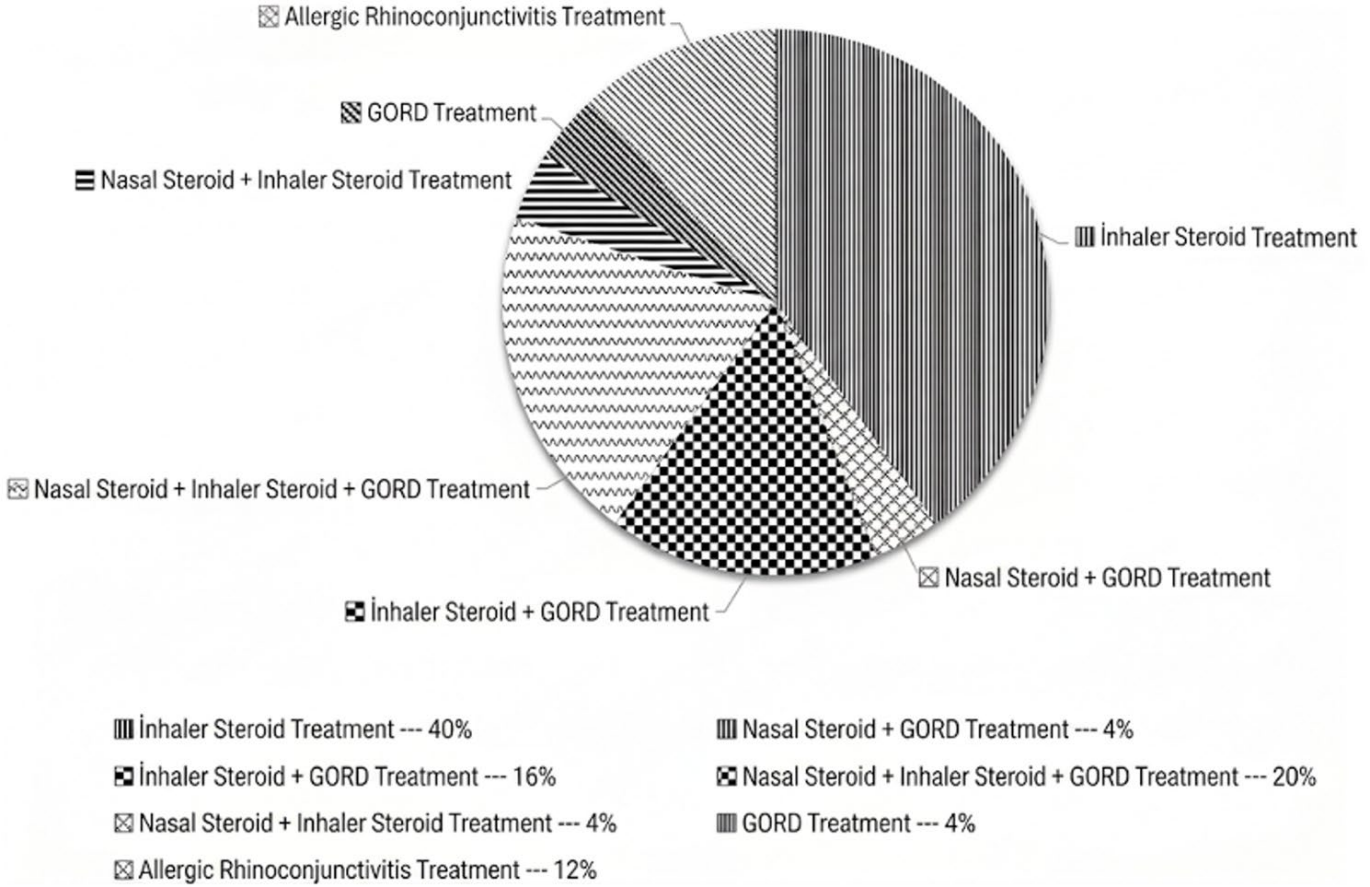
Distribution of treatment strategies administered over a 12-month follow-up in 69 children with recurrent croup

Treatment decisions were made individually for each patient based on their medical history, clinical findings, and laboratory results. Only inhaled corticosteroids were prescribed to 40% of the 69 patients included (*n* = 27). Nasal corticosteroids and gastroesophageal reflux disease (GERD) treatment were administered to 4% (*n* = 3), while inhaled corticosteroids and GERD treatment were administered to 16% (*n* = 11) (Fig. 2). Triple therapy consisting of nasal corticosteroids, inhaled corticosteroids, and GERD treatment was administered to 20% of patients (*n* = 14). Additionally, nasal and inhaled corticosteroids without GERD treatment were prescribed to 4% of patients (*n* = 3). GERD treatment alone was administered to 4% of patients (*n* = 3). Furthermore, specific treatments for allergic rhinoconjunctivitis were administered to 12% of patients (*n* = 8).

### Comparison of patients with and without recurrence of croup in the first 6 months

A comparative analysis was performed between cases with and without croup recurrence during the first 6 months of follow-up (Table 1). There was no significant difference in gender distribution between the two groups (males 59% and 41%; OR: 0.91; 95% CI: 0.33–2.54; *p* = 0.86). The mean age was similar between patients without recurrence (4.34 ± 1.54 years) and those with recurrence (4.54 ± 1.43 years) (*p* = 0.56). There was no significant difference in eosinophil count (328 ± 322 and 264 ± 307 cells/µL; *p* = 0.45) or total serum IgE levels (123 ± 361 and 254 ± 460 IU/mL; *p* = 0.25) between the groups. Nocturnal cough was significantly more common in patients with recurrent croup (53% vs. 47%; OR: 3.19; 95% CI: 1.15–8.91; *p* = 0.02). Exertional cough tended to be more common in recurrent croup (60% vs. 40%), but this difference was not statistically significant (OR: 2.76; 95% CI: 0.85–8.94; *p* = 0.08).

**Table 1:** Comparison of patients with and without croup recurrence during the first 6 months

		Without Croup Recurrence (*n* = 41)	With Croup Recurrence (*n* = 28)	Odds ratio	95% CI	*p*
Sex	Boys (%)	27 (59)	19 (41)	0.910	0.33–2.54	0.86 ^a^
Age	Years	4.34 (1.54)	4.54 (1.43)			0.56^b^
Eosinophil count	cells/µL*(SD)*	328 (322)	264 (307)			0.45^b^
Total IgE	IU/mL *(SD)*	123 (361)	254 (460)			0.25 ^b^
Nighttime cough	Yes *(%)*	19 (47)	15 (53)	3.19	1.15–8.91	0.02 ^a^
Exertional cough	Yes *(%)*	16 (40)	17 (60)	2.76	0.85–8.94	0.08 ^a^
wheezing	Yes *(%)*	25 (62)	11 (38)	0.890	0.26–3.09	0.86 ^a^
Preceding URTI	Yes *(%)*	22 (54)	13 (46)	1.45	0.54–3.89	0.46 ^a^
Nighttime feeding	Yes*(%)*	26 (56)	12 (44)	1.40	0.53–3.68	0.49 ^a^
Allergic rhinoconjunctivitis	Yes *(%)*	29 (70)	8 (30)	0.520	0.18–1.5	0.23 ^a^
Atopic dermatitis	Yes *(%)*	24 (58)	12 (42)	0.190	0.001–3.89	0.14 ^a^
Household tobacco exposure	Yes *(%)*	21 (52)	13 (48)	1.62	0.59–4.38	0.34 ^a^
Cesarean delivery	Yes *(%)*	24 (58)	12 (42)	1.15	0.42–3.12	0.78 ^a^
Birth time	Term *(%)*	23 (57)	12 (43)	0.260	0.03–2.42	0.21 ^a^
GERD diagnosis	Yes *(%)*	21 (50)	14 (50)	1.48	0.89–24.7	0.78 ^a^
Family history of atopy	Yes *(%)*	14 (33)	19 (67)	4.38	1.39–13.7	0.01 ^a^
Skin prick test	Positive *(%)*	23 (56)	12 (44)	1.19	0.38–3.67	0.76^a^

^a^ – Chi square test

^b^- Student t test

*NICU* Neonatal intensive care unit, *GERD* Gastroesophageal reflux disease, *URTI* Upper respiratory tract infection, *SD* Standard deviation

There were no significant differences between groups in terms of wheezing, previous upper respiratory tract infections (URTI), nighttime feeding, allergic rhinitis and conjunctivitis, atopic dermatitis, household tobacco exposure, cesarean deliveries, term births, previous PICU admissions, GERD diagnoses, or positive skin prick test results (all *p* > 0.05). A family history of atopy was strongly associated with recurrence and was observed more frequently in children who experienced recurrent croup attacks during follow-up (*p* = 0.01).

### Flexible optical bronchoscopy findings

Flexible optical bronchoscopy (FOB) was performed in seven children (10%) with persistent or atypical clinical features. In four children, laryngeal findings (posterior laryngeal erythema, interarytenoid edema, and increased secretions) suggestive of possible reflux-related irritation were recorded; objective reflux testing was not available and these findings are non-specific. Airway compression characterized by anterior tracheal collapse, pulsatile narrowing of the distal trachea, and dynamic expiratory collapse was detected in three children; thoracic CT angiography confirmed vascular ring anomalies in two cases and a tracheal hemangioma in one case.

The mean age of patients undergoing FOB was lower (3.0 ± 0.8 vs. 4.6 ± 1.5 years; *p* = 0.007), and they had experienced more frequent croup attacks prior to clinical follow-up (5.9 ± 3.4 vs. 3.7 ± 1.8; *p* = 0.008) and had higher total IgE levels (681 ± 772 vs. 127 ± 321 IU/mL; *p* = 0.003). In the small subgroup that underwent FOB, the high risk of bias due to indication should be considered as hypothesis-generating observations rather than definitive comparative data with the group that did not undergo FOB.

### Multivariable logistic regression analysis for croup recurrence in the first 6 and 12 months

**Fig. 3:**
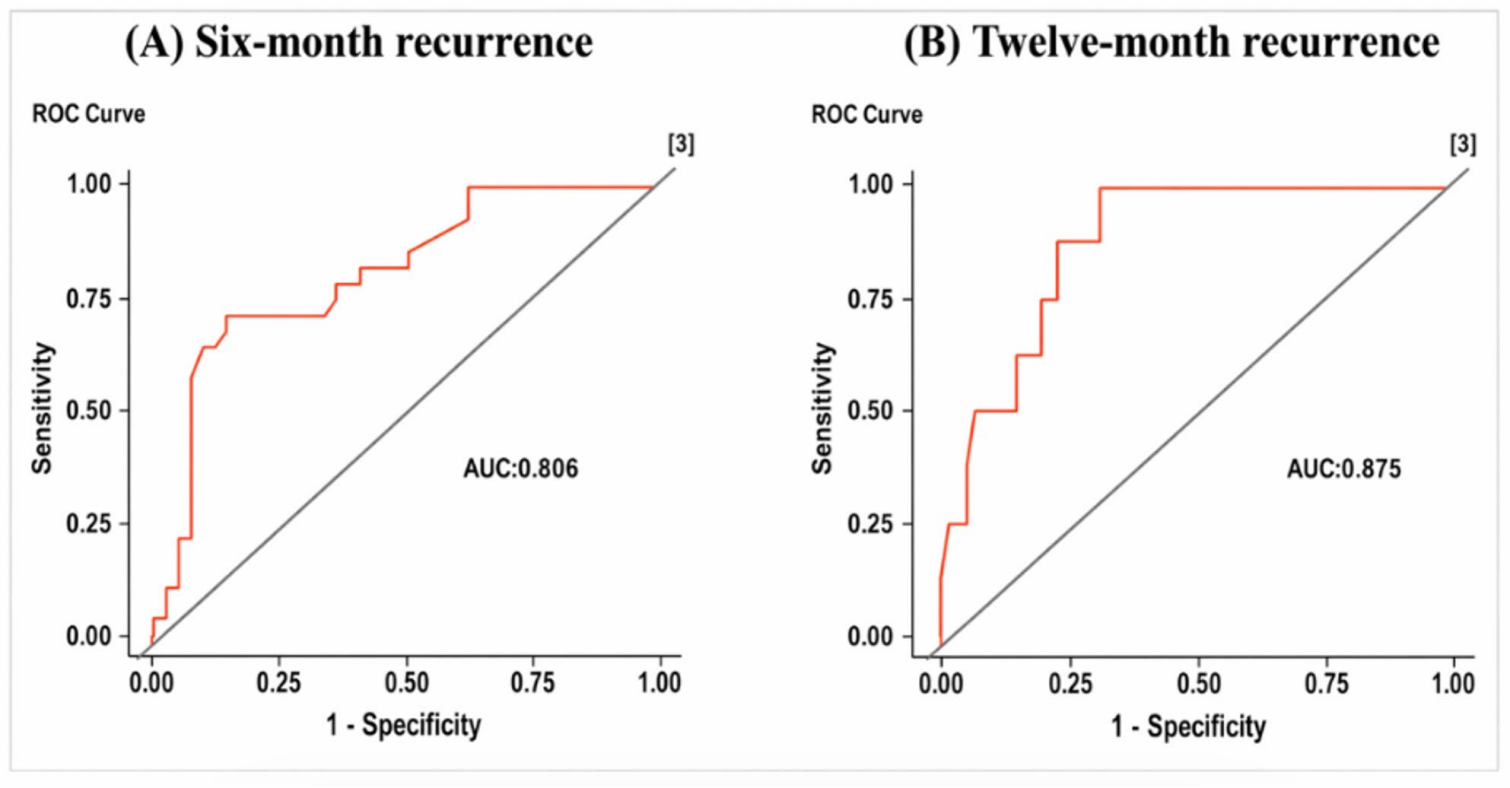
ROC curves for multivariable models predicting 6-month and 12-month recurrence. AUC, standard error (SE), and 95% confidence intervals (95% CI) were derived using the Hanley–McNeil method based on class counts

A binomial logistic regression model was constructed to identify independent predictors of croup recurrence during the first 6 months and 12 months of follow-up. Six-month recurrence. In the multivariable model, family history of atopy was independently associated with 6-month recurrence (aOR 7.50, 95% CI 1.96–28.68, *p* = 0.003). The number of croup episodes at presentation also predicted recurrence (aOR 1.43 per episode, 95% CI 1.06–1.92, *p* = 0.020), corresponding to ~ 43% higher odds for each additional episode. Night-time cough and exertional cough were not independently associated (aOR 2.17, 95% CI 0.59–8.00, *p* = 0.243; and aOR 3.04, 95% CI 0.73–12.70, *p* = 0.127, respectively). Age was not significant (aOR 1.28 per year, 95% CI 0.84–1.97, *p* = 0.250). Overall discrimination was good (AUC 0.806), and model fit indices were acceptable (Deviance 73.1, AIC 85.1, McFadden’s R² 0.215) (Fig. 3).

**Table:** 

Model	AUC	SE	95% CI
Six-month recurrence	0.806	0.056	0.696–0.916
Twelve-month recurrence	0.875	0.082	0.715–1.000

Twelve-month recurrence. In the multivariable model, family history of atopy was strongly associated with 12-month recurrence (aOR 9.11, 95% CI 1.12–74.00, *p* = 0.039). Age was protective (aOR 0.48 per year, 95% CI 0.24–0.95, *p* = 0.035). The number of croup episodes at presentation predicted 12-month recurrence (aOR 1.66 per episode, 95% CI 1.08–2.54, *p* = 0.020), indicating ~ 66% higher odds with each additional episode. Night-time cough and exertional cough were not independently associated (aOR 0.60, 95% CI 0.08–4.50, *p* = 0.621; and aOR 2.38, 95% CI 0.21–12.70, *p* = 0.484, respectively). Discrimination was excellent (AUC 0.875), and model fit indices were acceptable (Deviance 34.6, AIC 46.6, McFadden’s R² 0.300) (Fig. 3).

There were shared predictors across different time horizons. Family history of atopy and the number of croup episodes at presentation independently predicted recurrence at both 6 and 12 months, with similar directions and clinically meaningful effect sizes (aORs ~ 7–9 for atopy and ~ 1.4–1.7 per additional episode). Age showed a time-dependent pattern—non-significant at 6 months but protective at 12 months—while nighttime and exertional cough were not independently associated at either time point.

## Discussion

In this retrospective cohort study, we aimed to identify prognostic factors associated with further croup recurrences during a one-year follow-up period in children under six years of age diagnosed with recurrent croup. Our findings showed that a family history of atopy and a history of more frequent croup attacks prior to the initial evaluation were significant independent predictors of croup recurrence within the first six months. These results support evidence suggesting that recurrent croup is not solely caused by recurrent viral infections but may also be influenced by the child’s genetic makeup and predisposition to allergic conditions.

Previous studies have demonstrated a link between atopy and recurrent croup in family history and suggested that genetic or immune system factors may play a role in this condition [13, 14]. Atopy may make the airways more sensitive or cause more inflammation, which can lead to more frequent or longer-lasting respiratory problems in the upper airways [15]. In our cohort, the presence of a positive family history increased the likelihood of early recurrence by more than sevenfold, emphasizing the importance of allergen sensitivity screening and assessment of atopic comorbidity in recurrent cases. Additionally, in our study, the presence of frequent croup attacks prior to the first outpatient visit was found to be a significant predictor of croup recurrence. Previous findings suggest that anatomical, immunological, or inflammatory factors may reflect an innate susceptibility in croup recurrence [16]. Identifying children with early and recurrent attacks may allow for closer monitoring and evaluation of preventive treatments.

In univariate analyses, younger age, nighttime cough, and exertion cough were more frequently observed in cases with recurrent episodes; however, these variables did not remain statistically significant in multivariate models and should therefore be interpreted as exploratory findings. These non-significant trends may reflect developmental airway characteristics and increased sensitivity to triggers in young children, as suggested by previous studies on age-related changes in airway size and immune responses [3, 17].

Flexible optical bronchoscopy and thoracic CT were performed in a small, clinically selected subgroup with atypical or persistent symptoms. Laryngeal erythema/interarytenoid edema and increased secretions were recorded and were interpreted as possible reflux-related irritation; however, these findings are non-specific and cannot establish gastroesophageal reflux disease without objective reflux assessment (e.g., pH-impedance) [18–20]. Thoracic CT and thoracic CT angiography confirmed vascular ring anomalies and a tracheal hemangioma in selected cases. These descriptive findings support the role of advanced investigations when clinical features raise suspicion for structural disease, but our study was not designed to evaluate diagnostic yield or to derive imaging decision rules. In children with recurrent or atypical croup, the differential diagnosis may include structural airway lesions, vascular compression, gastroesophageal/laryngopharyngeal reflux, foreign body aspiration, recurrent respiratory papillomatosis, subglottic stenosis, primary ciliary dyskinesia, cystic fibrosis, and immunodeficiency. In our cohort, known chronic lung diseases such as cystic fibrosis and primary ciliary dyskinesia, as well as known immunodeficiencies, were excluded at study entry based on available clinical records.

Clinically, our findings suggest that children with a family history of atopy and a higher burden of prior episodes have a higher probability of further recurrences and may merit closer follow-up. These independent predictors are consistent with prior literature linking atopic background and episode burden with recurrent croup risk [16, 21, 22]. Importantly, our findings are hypothesis-generating and do not imply validated decision thresholds or a binary risk classification; external prospective validation is required before any clinical prediction tool can be recommended.

This study has several limitations. First, its retrospective design and single-center scope in a tertiary pediatric clinic may limit its generalizability and may have enriched our cohort with children who had more complex, severe, or atopic phenotypes. Second, the determination of outcomes, based on electronic medical records, relies on croup cases requiring medical care at our institution and documented by clinicians. Mild recurrences treated at home or in other hospitals were likely underreported, which may have led to non-differential misclassification and an underestimation of the true recurrence rate. Third, information on potential important determinants of upper respiratory tract infections and croup recurrence, such as systematic viral testing, influenza vaccination status, attendance at daycare or preschool, and exposure to outdoor air pollution, could not be consistently obtained from the charts, and we did not have standardized information on the seasonality of attacks. Finally, only a small, clinically selected subgroup underwent flexible optical bronchoscopy and CT; these investigations were performed for clinical indications, introducing strong indication bias. Laryngeal findings were non-specific and reflux was not confirmed with objective testing, so reflux-related interpretations should be considered exploratory. Moreover, our analyses did not assess whether predictor profiles modify the diagnostic yield or utility of advanced investigations, and we did not validate a risk score or decision thresholds. Despite these limitations, multivariable modeling identified two readily available clinical factors that were consistently associated with recurrence, providing a basis for prospective validation.

## Conclusion

Recurrent croup in young children is associated with identifiable clinical factors, particularly a family history of atopy and a higher number of prior croup episodes, which independently predict further recurrences over 6 and 12 months. These findings may help clinicians recognize children at higher probability of recurrence and support the design of prospective validation studies. Decisions about advanced investigations should remain driven by clinical features and current guidelines rather than by unvalidated risk thresholds.

## Data Availability

De-identified individual participant data, a data dictionary, and analysis code will be made available upon reasonable request to the corresponding author, subject to institutional data-sharing policies and Turkish data protection regulations. If feasible, anonymized summary data and code will be deposited in an open repository upon acceptance.

## Abbreviations

CT: Computed tomography
FOB: Flexible optical bronchoscopy
GERD: Gastroesophageal reflux disease
ICS: Inhaled corticosteroids
IgE: Immunoglobulin E
NICU: Neonatal intensive care unit
PICU: Pediatric intensive care unit
SPT: Skin prick test
URTI: Upper respiratory tract infection
